# Gender and Entrepreneurship in Pandemic Time: What Demands and What Resources? An Exploratory Study

**DOI:** 10.3389/fpsyg.2021.668875

**Published:** 2021-05-19

**Authors:** Silvia De Simone, Jessica Pileri, Max Rapp-Ricciardi, Barbara Barbieri

**Affiliations:** ^1^Department of Pedagogy, Psychology and Philosophy, University of Cagliari, Cagliari, Italy; ^2^Department of Psychology, University of Gothenburg, Gothenburg, Sweden; ^3^Department of Political and Social Sciences, University of Cagliari, Cagliari, Italy

**Keywords:** women entrepreneurs, JD-R model, entrepreneurial self-efficacy, work-family inter-face, entrepreneurial demands, elaborate and proactive social strategies, COVID-19

## Abstract

Due to the coronavirus disease 2019 (COVID-19) epidemic, global economies have suffered an exogenous shock never seen before with a strong economic and psychosocial impact on organizations. Italy, in the context of the research, has been severely affected. The economic crisis has mainly affected women. In this scenario, entrepreneurial perceived success (objective and subjective) is influenced by increasingly burdensome job demands that entrepreneurs have to face up. Using the job demand-resources model, the study aims to broaden the knowledge of the determinants of entrepreneurial perceived success in the current emergency moment. In particular, as regards of the demands, alongside the specific entrepreneurial demands (time demands, uncertainty and risk, and responsibility), we also decided to include the negative interface family–work in both directions from-family-to-work (NEGWIF) and from-work-to-family (NEGFIW). Regarding the resources, we considered entrepreneurial self-efficacy (researching, planning, marshaling, implementing people, and implementing financial), proactive and elaborate social strategies (SS), and both directions of the positive interface: from-family-to-work (POSWIF) and from-work-to-family (POSFIW). All participants are women entrepreneurs (*N* = 137) who have completed a self-report questionnaire. We explored the associations between demands, resources, and the dimensions of success through hierarchical regressions. As for the demands, time demands, uncertainty and risk, NEGWIF, and NEGFIW negatively influenced the perceived entrepreneurial success. Regarding resources, planning, implementing financial, proactive and elaborate SS positively influenced the perceived entrepreneurial success.

## Introduction

Businesses led by women play an increasingly strategic role in the Italian economy. In Italy (September 30, 2019), over 1.3 million enterprises were owned by women (1,340,580) (Osservatorio sull'imprenditoria femminile, 2020). In 2019, more than 10 million women were entrepreneurs in the European Union Member States (Koltai et al., 2020).

But due to the severe acute respiratory syndrome coronavirus 2 (SARS-CoV-2) discovered in the late 2019 (Zhu et al., 2020), global economies have undergone an exogenous shock never seen before (GDA, 2020). Italy has also been seriously affected (Remuzzi and Remuzzi, 2020). Therefore, the Italian government imposed a lockdown, in which there was a total closure of schools, public places, and most of the companies. This lockdown (phase 1) lasted about 2 months (from March to May, 2020). After this first measure, there was a phase defined “first gradual reopening,” in which one had to live with the virus by adopting specific security measures (phase 2, from May to June, 2020). This re-starting phase also included the reopening of businesses. But, according to the latest survey by Confindustria (2020), the main Italian business association, 97.2% of the companies suffered from a negative impact. Furthermore, 43.2% of the Italian companies reported very serious financial problems. In this scenario, the success of the Italian companies certainly is changing. This state of emergency has and still is generating a lot of pressure at the job, and entrepreneurs are facing up various obstacles and can rely only on their own resources. This situation particularly reflects the specific conditions of women entrepreneurs, who are utterly affected by the negative economic outcomes caused by the pandemic. Women entrepreneurs have greater problems with liquidity, procurement of supplies, greater difficulties related to the decline in employment, more constraints in access to credit, and technological problems, compared to their male counterparts (Unioncamere, 2021). In light of these problems, this article aims to study the potential entrepreneurial success factors in this time of crisis and restarting, taking job demands and job resources into consideration, with potentially interesting implications for interventions.

Traditionally, entrepreneurial success has been conceptualized through business growth or market dominance (Cooper et al., 1994; Van Praag and Versloot, 2007). Actually, the mere use of objective and financial parameters as a single measure of success has been criticized (Kuratko et al., 1997; Walker and Brown, 2004); this is because many subjective variables come into play in the meaning and representation of what it can be defined as “entrepreneurial success” (Simpson et al., 2004; Walker and Brown, 2004; Amato et al., 2017). Consequently, multiple subjective sources of success have been considered in the literature (e.g., personal satisfaction and flexibility, Walker and Brown 2004; family security, Kuratko et al., 1997; Shane et al., 2003). In this study, we took into consideration both the objective and subjective sources/perceptions of success: Firm performance and personal financial rewards as objective sources and workplace relationships and personal fulfillment as subjective ones. These dimensions have proved to be strategic for understanding entrepreneurial success (Wach et al., 2016).

To better understand the influences of the challenges due to the pandemic on business success, we propose the use of the job demands–resources model (JD-R model, Bakker and Demerouti, 2007) to try to clarify the position of women entrepreneurs in this particular historical moment of emergency and the effects on business success. This model postulates the simultaneous presence of job demands and job resources. Job resources “refer to those physical, psychological, social or organizational aspects of work which (1) reduce the demand for work and the related physiological and psychological costs; (2) are functional to the achievement of the work objectives; (3) stimulate personal growth, learning and development” (Schaufeli and Bakker, 2004, p. 296). The application of this model to entrepreneurs is present in the literature; however, although there is sufficient research on the application of the JD-R model to employees, studies on entrepreneurs are rather few (Dijkhuizen et al., 2016). This model argues that each profession can have its own specific risk factors. For this reason, in this study, we use specific resources and demands related to entrepreneurial work and other types of resources and demands that can help us to better understand the dynamics between demands and resources affecting women entrepreneurs in this particular pandemic time.

Based on earlier discussion, the study aims to explore entrepreneurial resources and demands in pandemic time and their influence on the success of women entrepreneurs. The contribution offered to the literature on this field concerns both the understanding of what entrepreneurial resources and needs are in this period of emergency and practical implications. Based on the findings, potential strategies are discussed to improve women's entrepreneurial success.

### Job Demands and Success

Job demands refer to “those physical, psychological, social, or organizational aspects of the job that require sustained physical and/or psychological (cognitive and emotional) effort and are therefore associated with certain physiological and/or psychological costs” (Schaufeli and Bakker, 2004, p. 296). In an exploratory way, we have chosen the demands that could influence the current job demands for entrepreneurs during the pandemic. We have selected both non-specific demands and specific demands for entrepreneurs. The following demands have been identified as the characterizing dimensions of entrepreneurial work: time demands, uncertainty and risk, and responsibility (Dijkhuizen et al., 2014). Furthermore, in the pandemic situation, these demands certainly have increased. In times of economic crisis, it is reasonable to think that entrepreneurs must be more available and devote more time to business management. They must face greater uncertainties and risks and increase the sense of responsibility toward their company to ensure its survival. These specific dimensions are stronger predictors than the other non-specific dimensions for entrepreneurs (Dijkhuizen et al., 2014, 2016). Alongside to these specific entrepreneurial demands mentioned above, we decided to also include the family–work conflict. The reason is that the lockdown in Italy due to coronavirus disease 2019 (COVID-19) also led to the closure of schools and the suspension of other assistance services for families and individuals. In Italy, it is very important to examine the work–family interface since Italian women dedicate 2h01' per day to housework and taking care of the family (caring for the children, the elder, and cohabitants with disabilities) while men spend 1h24'. And if one would consider parenting (in families with minor children), the participation rate of fathers is 46.8% while that of the mothers is 73% (Istat, 2019). Italian women, along with the Romanian women, spend more time in such activities than women in any other country within the European community (5h02'). Italian and Greek men, on the other hand, are those who dedicate <2 h a day for unpaid work at home, which shows a huge gender gap (3h08') (*ibidem*). In this difficult pandemic situation, the request for the participation in multiple roles (work and family roles) could increase. Role theory (Biddle, 1986) has been used to explain strategies for reconciling family and work life, assuming that there are limited resources (e.g., Rothbard, 2001), based on the assumption that a greater number of roles required more effort to balance them generating interference (Hsu et al., 2016). The experience of work–family conflict can occur in both directions from work-to-family as well as from family-to-work, and these have been established as distinct constructs (Grzywacz and Marks, 2000). Negative interface from-work-to-family (NEGWIF) is recognized as a form of conflict between the roles in which general needs and work needs interfere with the performance of family-related responsibilities. Negative interface from-family-to-work (NEGFIW), on the other hand, is a form of conflict between the roles in which the general needs and demands of the family domain interfere with the performance of job-related responsibilities (Netemeyer et al., 1996). The work–family conflict has not been studied extensively in relation to entrepreneurs (Jennings and McDougald, 2007). Little attention was paid to women entrepreneurs (Poggesi et al., 2019) in relation to the work–family interface. In fact, women generally experience higher levels of family–work conflict than men (Fahlén, 2014; Lee et al., 2014). This trend is even greater when it comes to women entrepreneurs (Loscocco et al., 1991). In addition, women entrepreneurs can have multiple roles in the family and in their company, which could lead to role conflicts if poorly managed. In general, women entrepreneurs have more responsibilities and work more hours than employed women, having more difficulties in balancing life and work (Kim and Ling, 2001; DeMartino et al., 2006; De Simone and Priola, 2015). Women entrepreneurs need to become “super(wo)men,” autonomous, and agentic and need to keep control over all aspects of their life (work, house, etc.), to successfully manage both work and family (De Simone and Priola, in press). For these reasons, we have decided to integrate, among the demand's dimensions, the family–work conflict (in both directions), to take into account also this aspect which is generally not considered, but which could be crucial for women entrepreneurs. Furthermore, in this time in which the demands have increased due to the pandemic, it could be difficult to involve adequate resources for balancing family roles and job roles in addition; it has been found that a high work–family conflict damages both perceived and objective entrepreneurial performances (Shelton et al., 2008).

Based on these premises, we hypothesize that:

H1: Demands (time demands, uncertainty and risk, responsibility, NEGWIF, and NEGFIW) are negatively related to the dimensions of the perceived entrepreneurial success (firm performance, personal financial rewards, workplace relationships, and personal fulfillment).

### Job Resources and Success

Concerning the resources, we have considered specifically personal resources. Personal resources are aspects of the self, related to resilience and to the individual sense of one's ability to successfully control the environment (Hobfoll et al., 2003). In this regard, we refer to those salient personal resources able to manage the current moment of crisis. In the literature, there is a growing attention that extends the JD-R model by integrating personal resources (e.g., self-efficacy and optimism: Xanthopoulou et al., 2007). Thus, in this study, we considered self-efficacy as a specific resource for entrepreneurs (this is because self-efficacy should be considered as specific to the situation, Bandura, 1997) and explored effectiveness in various dimensions related to entrepreneurial work (searching, planning, marshaling, implementing people, and implementing financial). These dimensions are also particularly useful for dealing with the current pandemic situation. We have considered the self-efficacy in all phases of the entrepreneur's work, from research to maintenance, because in this crisis moment caused by the pandemic, many entrepreneurs are forced to reinvent themselves as well as to resist in the market. Furthermore, SS are also useful for achieving one's goals (Guirdham, 1990). SS can be considered as the individual owner behavioral strategies (in contrast to firm strategies) (Von Gelderen et al., 2000). Zhao et al. (2010) and claim that SS are the behavioral plans applied to social interactions to achieve goals. Indeed, individuals use SS to deal with challenging social circumstances (Nurmi et al., 1997), such as the current pandemic situation. For these reasons, the use of elaborate and proactive SS can be decisive for entrepreneurial success. As an additional resource, we have included the possibility that the family context can also act as a resource and enrich the work of women entrepreneurs. In fact, some studies have highlighted the positive side of the family–work interface (e.g., Grzywacz and Butler, 2005), which embraces the idea of enrichment that could play a decisive role in this economic crisis that led to more active participation both in the family domain and in the work domain. The vast literature on this topic states that the coexistence with job roles can also be considered positive (e.g., Frone, 2003; Greenhaus and Powell, 2003; Grzywacz and Butler, 2005; Hill, 2005; Carlson et al., 2006). This approach assumes that participation in multiple roles is a resource that improves other areas of life (Barnett, 1998). This generates positive effects that have been called “positive spillover” (Barnett, 1998; Grzywacz and Marks, 2000), “enrichment” (Rothbard, 2001; Greenhaus and Powell, 2003), and “facilitation” (Frone, 2003; Grzywacz and Butler, 2005; Hill, 2005). Along this line, we also embrace the idea that the various social and psychological resources brought into play by the multiple roles of life are sources of empowerment (Ruderman et al., 2002). In particular, we consider both the positive interface from-work-to-family (POSWIF) and the positive interface from-family-to-work (POSFIW) to explore the mutually beneficial relationship between work and family. Women entrepreneurs are particularly benefited from the affective work–family enrichment and family-derived enrichment (Powell and Eddleston, 2011). So, enrichment could play a decisive role in this moment of crisis that led to more active participation both in the family domain and in the work domain.

Based on these premises, we hypothesize that:

H2: Resources (searching, planning, marshaling, implementing people, implementing financial, POSWIF, POSFIW, and proactive and elaborate SS) are positively related with perceived entrepreneurial success (firm performance, personal financial rewards, workplace relationships, and personal fulfillment).

## Materials and Methods

### Participants and Procedures

This study has involved the Italian women entrepreneurs during the pandemic second phase (or the phase of “gradual reopening”). The participants were recruited through entrepreneurial trade associations as AIDDA (Italian Association of Women Entrepreneurs and Corporate Executives) and Confindustria (General Confederation of Italian Industry). An anonymized questionnaire was presented to participants using two methods: online and on paper. There was no difference between the paper and online administration regarding the content and format of the questionnaire. A total of 159 women entrepreneurs aged 23–66 years (M = 43, SD = 11.09) are participated in the study. The firms of the entrepreneurs were mainly micro (<10 employees, 80.8%), small (10–50 employees, 9.6%), and medium (50–250 employees, 10%) sized enterprises, operating in a variety of sectors. As for the business sector, 11.3% have a company operating in the agricultural sector, 11.3% deal with crafts, 13.2% deal with catering, 37.7% deal with trade, and the remaining 26.4% deal with welfare services. The average age of the company was 25 years, ranging from 1 year to 82 years. Regarding the education level, 42.8% had a high-school diploma, 33.4% completed a bachelor's or master's degree, 8.3% completed a postgraduate specialization or a PhD course, and 15.5% qualified lower than a diploma level. In the sample, 51% of the participants were married, 10.7% lived with partner, 10.7% had a partner, 6% were divorced, 15.5% were single, and the remaining 6% were widowed. Participants were also asked to indicate the presence or absence of sons and/or daughters: 41.7% have no sons and/or daughters, while the remaining 58.2% have sons and/or daughters, and the average of the number of children is 2, ranging from 1 to 4.

### Measures

#### Work–Family Interface

Work and family dimensions were assessed through 14 items measuring 4 different theoretical dimensions: the NEGWIF (4 item, example: The demands of your job interfere with your home and family life? α = 0.982), the NEGFIW (4 item, example: The demands of your family or spouse/partner interfere with your work-related activities? α = 0.841), the POSWIF (3 item, example: You manage your time at home more efficiently as a result of the way you do your job? α = 0.747), and the POSFIW (3 item, example: You manage your time at work more efficiently because at home you have to do that as well? α = 0.635) (Kinnunen et al., 2006). This instrument has been validated for the Italian context (De Simone et al., 2018). Response categories for all of the items ranged from 1 (“never”) to 5 (“very often”).

#### Entrepreneurial Demands

We used the three scales specifically built for entrepreneurs developed by Dijkhuizen et al. (2014). The first scale is time demands (5 items, example: “Does it feel as if you have to be available for your company 24 h a day?” α = 0.846). The second scale is uncertainty and risk (6 items, example: “Do you find it hard to handle risks concerning your company?” α = 0.688). The third scale is responsibility (3 items, example: “Do you feel yourself 100% responsible for the satisfaction of the customers of your company?” α = 0.666). We used a 4-point Likert scale (never/always). Accuracy translation for the Italian context has been verified through back translation.

#### Entrepreneurial Self-Efficacy

To perform this measure, we used the scale developed by McGee et al. (2009). Entrepreneurial self-efficacy dimensions were assessed through 19 items measuring five different theoretical dimensions. The searching dimension investigates self-efficacy in developing an idea or identifying opportunities (item example: How much confidence do you have in your ability to design a product or service that will satisfy customer needs and wants? α = 0.858). Planning dimension measures self-efficacy in converting the initial idea into a business plan (item example: How much confidence do you have in your ability to design an effective marketing/advertising campaign for a new product or service? α = 0.847). The marshaling dimension measures self-efficacy in assembling resources to achieve the enterprise (item example: How much confidence do you have in your ability to clearly and concisely explain verbally/in writing my business idea in everyday terms? α = 0.827). The implementing financial dimension measures self-efficacy in making business (item example: How much confidence do you have in your ability to organize and maintain the financial records of my business? α = 0.870). The implementing people dimension refers to self-efficacy related to staff development (item example: How much confidence do you have in your ability to inspire, encourage, and motivate your employees? α = 0.866). Translation accuracy for the Italian context has been verified through back translation.

#### Proactive and Elaborate Social Strategies

We used the scale developed by Zhao et al. (2010). The scale consists in 10 items that measure the proactive and elaborate SS (item example: I actively improve my interpersonal skills, α = 0.852). We used a 5-point Likert response scale (1 definitely not like me, 5 exactly like me). Translation accuracy for the Italian context has been verified through back translation.

#### Entrepreneurial Success

Entrepreneurial success was assessed through 14 items measuring four different theoretical dimensions. We used the four scales developed by Wach et al. (2016). The firm performance dimension includes success criteria related to firm economic performance (example: Profit growth, α = 0.895). The workplace relationships dimension captures success definitions related to relationships with stakeholders within and outside the firm (example: Employee satisfaction, α = 0.777). The personal fulfillment dimension encompasses the personal aspects of success (example: Personal development, α = 0.608). The personal financial reward captures the desire for high income, that is, extrinsic rewards (example: Personal financial security, α = 0.918). We used a 5-point Likert response scale. Translation accuracy for the Italian context has been verified through back translation.

### Data Analyses

Descriptive statistical analyses were conducted. Preliminarily, Pearson's correlations were carried out. To examine the relationships between the demands and resources in the JD-R model on perceived entrepreneurial success dimensions, four hierarchical regressions were conducted. Predictor variables were entered into the regression equation in three blocks. In the first step (Model 1), we inserted the socio-demographic variables (age, educational level, and number of sons/daughters); in the second step (Model 2), we added, as a predictors, demands (time demands, uncertainty and risk, responsibility, NEGWIF, and NEGFIW); and finally, in the third step (Model 3), we added resources (searching, planning, marshaling, implementing people, implementing financial, POSWIF, POSFIW, and proactive and elaborate SS) as predictors.

## Results

Pearson's correlations suggested correlations between several predictors and the outcome variables (see Table 1). Firm performance was correlated with uncertainty and risk (*r* = −0.25, *p* < 0.05), NEGWIF (*r* = 0.32, *p* < 0.01), NEGFIW (*r* = 0.26, *p* < 0.05), searching (*r* = 0.42, *p* < 0.01), planning (*r* = 0.40, p <0.01), marshaling (*r* = 0.40, *p* < 0.01), implementing people (*r* = 0.32, *p* < 0.01), POSWIF (*r* = 0.29, *p* < 0.01), POSFIW (*r* = 0.22, *p* < 0.01), and proactive and elaborate SS (*r* = 0.39, *p* < 0.01). Personal financial reward was correlated with uncertainty and risk (*r* = −0.35, *p* < 0.01), searching (*r* = 0.37, *p* < 0.001), planning (*r* = 0.48, *p* < 0.01), marshaling (*r* = 0.38, *p* < 0.001), implementing people (*r* = 0.29, *p* < 0.01), and proactive and elaborate SS (*r* = 0.39, *p* < 0.01). Workplace relationships were correlated with time demands (*r* = 0.51, *p* < 0.01), responsibility (*r* = 0.26, *p* < 0.05), NEGWIF (*r* = 0.41, *p* < 0.01), NEGFIW (*r* = 0.41, *p* < 0.01), searching (*r* = 0.37, *p* < 0.01), planning (*r* = 0.35, *p* < 0.01), marshaling (*r* = 0.42, *p* < 0.01), implementing people (*r* = 0.41, *p* < 0.001), implementing financial (*r* = −0.23, *p* < 0.05), POSWIF (*r* = 0.41, *p* < 0.01), POSFIW (*r* = 0.44, *p* < 0.01), and proactive and elaborate SS (*r* = 0.51, *p* < 0.01). Finally, personal fulfillment was correlated with uncertainty and risk (*r* = −0.49, *p* < 0.01), NEGWIF (*r* = -−0.29, *p* < 0.01), NEGFIW (*r* = −0.26, *p* < 0.05), planning (*r* = −0.31, *p* < 0.01), implementing people (*r* = −0.25, *p* < 0.05), and proactive and elaborate SS (*r* = 0.33, *p* < 0.01).

**Table 1:** Correlations between variables.

	**M**	**SD**	**1**	**2**	**3**	**4**	**5**	**6**	**7**	**8**	**9**	**10**	**11**	**13**	**14**	**15**	**16**
Time demands	2.95	0.72	1														
Uncertainty and risk	1.93	0.43	0.20	1													
Responsibility	3.34	0.64	0.49^**^	0.05	1												
NEGWIF	3.53	1.37	0.36^**^	0.10	0.24^*^	1											
NEGFIW	2.65	1.06	0.37^**^	0.20	0.35^**^	0.71^**^	1										
Searching	3.88	0.84	0.47^**^	−0.08	0.23^*^	0.44^**^	0.36^**^	1									
Planning	3.71	0.76	0.50^**^	−0.15	0.20	0.27^*^	0.23^*^	0.70^**^	1								
Marshaling	3.86	0.76	0.46^**^	−0.24^*^	0.31^**^	0.24^*^	0.37^**^	0.62^**^	0.62^**^	1							
Implementing people	3.8	0.74	0.48^**^	−0.29^**^	0.34^**^	0.35^**^	0.30^**^	0.59^**^	0.48^**^	0.72^**^	1						
Implementing financial	3.13	0.84	−0.27^*^	0.27^*^	−0.15	−0.15	−0.14	−0.30^**^	−0.35^**^	−0.50^**^	−0.52^**^	1					
Proactive and elaborate social strategies	3.33	0.70	0.31^**^	−0.06	0.23^*^	0.31^**^	0.48^**^	0.41^**^	0.16	0.37^**^	0.33^**^	−0.17	1				
POSWIF	3.67	1.01	0.35^**^	0.21^*^	0.33^**^	0.46^**^	0.63^**^	0.36^**^	0.14	0.20	0.23^*^	−0.07	0.58^**^				
POSFIW	3.75	1.06	0.34^**^	−0.16	0.19	0.31^**^	0.38^**^	0.53^**^	0.37^**^	0.50^**^	0.42^**^	−0.38^**^	0.52^**^	1			
Firm performance	3.46	0.90	0.20	−0.25^*^	0.17	0.32^**^	0.26^*^	0.42^**^	0.40^**^	0.40^**^	0.32^**^	−0.15	0.29^**^	0.39^**^	1		
Personal financial rewards	3.16	0.92	0.17	−0.35^**^	0.06	0.15	0.09	0.37^**^	0.48^**^	0.38^**^	0.29^**^	−0.09	0.18	0.39^**^	0.86^**^	1	
Workplace relationships	4.13	0.55	0.51^**^	−0.00	0.26^*^	0.41^**^	0.41^**^	0.37^**^	0.35^**^	0.42^**^	0.41^**^	−0.23^*^	0.41^**^	0.55^**^	0.61^**^	0.51^**^	1
Personal fulfillment	3.70	0.55	0.09	−0.49^**^	−0.02	−0.29^**^	−0.26^*^	0.18	0.31^**^	0.20	0.25^*^	−0.19	0.01	0.25^*^	0.33^**^	0.47^**^	0.27^*^

* *p <0.05*;

** *p <0.01*;

*** *p <0.001*.

The results of the hierarchical regressions are shown in Table 2.

**Table 2:** Results of hierarchical regression analysis.

	**Firm performance**			**Personal financial rewards**			**Workplace relationships**			**Personal fulfillment**		
	*****β*****			**B**			*****β*****			**B**		
**Step 1: demographics**												
Age	−0.080	0.008	−0.006	0.051	0.076	0.071	−0.179	−0.134	−0.156	0.212	0.026	0.533
Educational level	0.119	0.422^**^	0.352^*^	0.150	0.405^**^	0.365^**^	−0.199	0.028	0.037	0.085	0.212	0.740
Number of sons/daughters	−0.018	−0.163	−0.187	−0.052	−0.091	−0.060	0.284^*^	0.105	0.258	−0.243	0.085	0.929
*R*^2^ Adj	−0.047			−0.038			0.093			0.045		
**Step 2: demands**												
Time demands		0.507^**^	0.306		0.636^**^	0.181		0.598^**^	0.601^*^		−0.243^*^	0.092
Uncertainty and risk		−0.441^**^	−0.255		−0.589^**^	−0.348^**^		−0.248	−0.102		−0.623^***^	−0.480^**^
Responsibility		0.078	0.032		0.010	0.005		0.056	−0.035		−0.067	−0.060
NEGWIF		0.626^**^	0.575^*^		0.487^*^	0.531^**^		0.306	0.183		−0.514^**^	−0.566^**^
NEGFIW		−0.370	−0.671^*^		−0.480^*^	−0.896^***^		−0.233	−0.528^*^		−0.027	−0.208
*R*^2^ Adj	0.382			0.404			0.362			0.583		
**Step 3: resources**												
Searching			−0.127			−0.289			−0.244			0.013
Planning			0.161			0.582^**^			−0.198			0.188
Marshaling			0.036			0.008			0.263			−0.101
Implementing people			0.325			0.297			−0.108			0.189
Implementing financial			0.375^*^			0.409^**^			−0.014			0.142
POSWIF			0.249			0.257			0.128			0.038
POSFIW			0.040			0.120			0.140			0.060
Proactive and elaborate social strategies			0.275			0.378^**^			0.596^**^			0.336^*^
*R*^2^ Adj	0.436			0.723			0.562			0.657		

* *p <0.05*;

** *p <0.01*;

*** *p <0.001*.

Model 3 was significant in all four regressions (firm performance *F* = 3,275, *p* < 0.01, personal financial rewards *F* = 8,671, *p* < 0.001, workplace relationships *F* = 4.765, *p* < 0.001, personal fulfillment *F* = 7.569, *p* < 0.001). The results of the hierarchical regression analysis suggested that NEGWIF (β = 0.575, *p* < 0.05), NEGFIW (β = −0.671, *p* < 0.05), and implementing financial (β = 0.375, *p* < 0.05) depicted significant relationships with firm performance. Uncertainty and risk (β = −0.348, *p* < 0.01), NEGWIF (β = 0.531, *p* < 0.01), NEGFIW (β = −0.896, *p* < 0.001), planning (β = 0.582, *p* < 0.01), implementing financial (β = 0.409, *p* < 0.001), and proactive and elaborate SS (β = 0.378, *p* < 0.01) depicted significant relationships with personal financial rewards. Time demands (β = 0.601, *p* < 0.05), NEGFIW (β = −0.528, *p* < 0.05), and proactive and elaborate SS (β = 0.596, *p* < 0.001) depicted significant relationships with workplace relationships. Finally, uncertainty and risk (β = −0.480, *p* < 0.001), NEGWIF (β = −0.566, *p* < 0.01), and proactive and elaborate SS (β = 0.336, *p* < 0.05) depicted significant relationships with personal fulfillment.

## Discussion

The principal objective of the study was to explore the relationships between the demands and resources considered and the perceived entrepreneurial success by women entrepreneurs in pandemic time. In particular, we wanted to explore the relationship between some demands like entrepreneurial demands (time demands, uncertainty and risk, and responsibility) and work–family conflict (NEGWIF and NEGFIW), some resources like entrepreneurial self-efficacy (searching, planning, marshaling, implementing people, and implementing financial), work–family enrichment (POSWIF and POSFIW), proactive and elaborate SS, and four dimensions of perceived entrepreneurial success (firm performance, personal financial rewards, workplace relationships, and personal fulfillment). The results partially confirmed the relationships hypothesized.

Regarding the firm performance, among demands, NEGFIW showed a negative relationship as hypothesized. NEGWIF instead showed a positive relationship. During the pandemic time, women entrepreneurs felt particularly pressured by requests from the family and work domains. The data showed that the participants perceive that when work negatively interferes with the family, the chances of entrepreneurial success in relation to firm performance increase. On the contrary, when the family interferes negatively with work, the perception of success in terms of firm performance decreases. In other words, the interviewed women entrepreneurs seemed to have a clear idea of the need to sacrifice the family and to give priority to work for entrepreneurial success in terms of firm performance. As stated by Hall (1990), it is likely that feelings about how successful one is in balancing work and family may come from situations that represent a sacrifice of one domain for the other. Women entrepreneurs, consistent with neoliberal ideals, mainly invest in the market and sacrifice the family for the business (De Simone and Priola, in press). The successful businesswomen are “women heroines” characterized by confidence, control, and courage to face and overcome gendered barriers (Adamson and Kelan, 2019). Perhaps this is even more evident because the current pandemic crisis increased the workload of women, both in their occupation and in their housework (Del Boca et al., 2020) asking them to be superwomen at work and in the family context. More time spent on working, neglecting the family, leads to an even greater investment in work, betting on economic growth determined precisely by the time spent at work. Women experience greater conflict between work and family roles than men (e.g., Noor, 2004; Welter, 2004), and given the results obtained, especially in relation to personal fulfillment, the pandemic period could aggravate this gap. With respect to resources, the “implementing financial” dimension is a key factor in predicting success in terms of firm performance. This is in line with the literature that has found relationships between self-efficacy in making business grow and firm performance of women entrepreneurs (e.g., Asandimitra and Kautsar, 2017).

Concerning personal financial rewards, among demands, uncertainty and risk showed a negative relationship as hypothesized. Among entrepreneurial demands, uncertainty and risk in this pandemic scenario is the dimension that interests personal financial rewards of women entrepreneurs. Likewise to the results related to firm performance, NEGFIW showed a negative relationship as hypothesized, but NEGWIF instead showed a positive relationship. The conflict between family and work plays a key role in explaining the entrepreneurial success also in relation to personal financial reward: by sacrificing the family for work, the time spent on working increases, and hypothetically, the personal financial rewards grow. Regarding resources, the “implementing financial” and the “planning” dimensions of entrepreneurial self-efficacy also predicted personal financial rewards. In this case, even proactive and elaborate SS maintain a significant positive relationship with personal financial rewards. Probably, the perception of knowing how to act appropriately in social contexts can lead women to create more networks that can be fruitful. The SS are behavioral strategies of the individual owner (in contrast to firm strategies) (Von Gelderen et al., 2000). For this reason, they are probably predictive of personal financial rewards (as an individual dimension) and not predictive of firm performance.

Regarding the workplace relationships, among demands, NEGFIW showed a negative relationship as hypothesized. Contrary to what we have hypothesized, among entrepreneurial demands, time demands dimension showed a positive relationship. One possible explanation may be that greater time pressure can lead to a greater sense of involvement that improves relationships at work. It could also happen that the increasing time spent at work means spending more time with people inside and outside the company, thus improving relationships. This is what may have happened to the entrepreneurs interviewed during the pandemic period, who, in order to save their company, have dedicated more time and more energy to the business and to relationships with employees. In addition, proactive and elaborate SS, as expected, also improve relationships at work. Proactive and elaborate SS ensure that social skills are continually improved (Zhao et al., 2010), and therefore, women entrepreneurs could use them for internal and external working relationships.

Finally, personal fulfillment among demands showed a negative relationship with uncertainty and risk and NEGWIF as we hypothesized. It is noteworthy that this is the only time NEGFIW was found to be negatively related to one of the dimensions of perceived entrepreneurial success. According to the findings, interference from work at family increases more work-related dimensions but decreases the levels of perceived entrepreneurial success related to the personal aspects of success. It would seem that personal fulfillment is to be sacrificed to increase the perceptions of the financial aspects of success. This can be understood in a penalizing way if we consider that work and family are intertwined areas for women entrepreneurs (Loscocco and Bird, 2012; Peris-Ortiz et al., 2012). The studies on the work–family interface in women entrepreneurs have investigated the work and family demands as sources of conflict and/or as a positive challenge (e.g., Bruni et al., 2004; Ahl, 2006). Mitigating work–family conflict is a strategy to handle the gender roles and manage the work–family conflict developing entrepreneurial business (Shelton, 2006).

Referring to resources, proactive and elaborate SS, as expected, also improve personal fulfillment. SS proved to be an important element in our study. Previous studies (e.g., Brush et al., 2005; Bogren et al., 2013) have reported that establishing good relationships is a key factor in influencing the success of women entrepreneurs. It should also be noted that among the entrepreneurial demands, responsibility was the only dimension not associated with any dependent variable. It therefore seems that this dimension does not affect the entrepreneurial success perceived during the pandemic period. Not even some dimensions of entrepreneurial self-efficacy have shown significant associations. Only financial and planning ability can help women entrepreneurs in the pandemic situation. Unexpectedly, not even the family–work enrichment is predictive of success. The dimension of the conflict between family and work prevails as a demand, and the possible enrichment between these two domains as a resource does not emerge. A possible explanation could lie in the fact that in the pandemic time in which the working life and private life have occupied the same times and (sometimes) the same spaces, it was difficult to create the POSFIW and the POSWIF and to think about the mutual exchange of the two domains in association with entrepreneurial success.

## Conclusion

The conflict between work and family domains plays an important role in the perceived entrepreneurial success. The work–family conflict affects all four dimensions of the perceived entrepreneurial success, proving to be a key element for the perception of the success of women entrepreneurs in this moment of pandemic. Difficulties related to risk management and uncertainty in managing one's own company, especially in this pandemic crisis, can negatively influence the perceived entrepreneurial access in terms of personal financial rewards and fulfillment. Time demands, counter intuitively in this moment instead, increase the success in relationships at workplace, probably due to the greater involvement they cause. The resources used are specific resources, which include self-efficacy in terms of planning and implementing financial. The perceived self-efficacy in transforming opportunities into business and in the ability to grow the company also from a financial point of view plays a decisive role. Proactive and elaborate SS influence three of the four dimensions of perceived entrepreneurial success, showing that interpersonal resources are a decisive factor for women entrepreneurs in this time of crisis.

## Practical Implications

The results show the key role of the family–work conflict on entrepreneurial success. The study focused on small businesses with limited resources, where generally women entrepreneurs are the main decision-maker and manager. Initiatives based on more favorable legislation to facilitate women entrepreneurs should be integrated by several actions aimed at implementing and diversifying childcare supply and specific family-friendly policies designed for women entrepreneurs. More services should be implemented for women entrepreneurs in this pandemic situation. Women entrepreneurs seem to feel the negative influence of uncertainty and risk. The political decisions of the Italian government should take into account the greater unpredictability given by the pandemic situation to create measures to protect companies that can guarantee a perception of greater stability over time. Entrepreneurial self-efficacy in the dimensions of planning and implementing financial has proved decisive as a resource to be used in the current situation; specific training can be implemented for women entrepreneurs. The ability to create and maintain networks, through proactive and elaborate SS, proved decisive. The possibility of creating networks and associations must be considered as a possible strategy to help women entrepreneurs in this particular crisis situation.

## Limitations

This is an exploratory study that provides some initial evidence on factors that could affect women's entrepreneurial success. However, this study has several limitations. First, this study used a cross-sectional design and self-reporting tools. Furthermore, the sample is certainly limited and not representative, not taking into account the different characteristics of the companies (size, sector, years of activity, etc.). Thus, this could limit the generalizability of the findings and have to be taken into account when interpreting results.

## Data Availability Statement

The raw data supporting the conclusions of this article will be made available by the authors, without undue reservation.

## Ethics Statement

Ethical review and approval was not required for the study on human participants in accordance with the local legislation and institutional requirements. The patients/participants provided their written informed consent to participate in this study.

## Author Contributions

JP, SD, and BB developed the research project and reviewed the literature. JP carried out the data analysis. MR-R reviewed the manuscript. All authors contributed to the article and approved the submitted version.

## Conflict of Interest

The authors declare that the research was conducted in the absence of any commercial or financial relationships that could be construed as a potential conflict of interest.

## References

[B1] Adamson M. Kelan E. (2019). Female heroes: celebrity executives as postfeminist role models. Br. J. Manage. 30, 981–996. 10.1111/1467-8551.12320

[B2] Ahl H. (2006). Why research on women entrepreneurs needs new directions. Entrepreneurship Theory Practice 30, 595–621. 10.1111/j.1540-6520.2006.00138.x

[B3] Amato C. Baron R. A. Barbieri B. Belanger J. J. Pierro A. (2017). Regulatory modes and entrepreneurship: the mediational role of alertness in small business success. J. Small Business Manage. 55, 27–42. 10.1111/jsbm.12255

[B4] Asandimitra N. Kautsar A. (2017). Financial self-efficacy on women entrepreneurs success. Int. J. Acad. Res. Business Soc. Sci. 7:3459. 10.6007/IJARBSS/v7-i11/3459

[B5] Bakker A. B. Demerouti E. (2007). The job demands-resources model: state of the art. J. Manage. Psychol. 22, 309–328. 10.1108/02683940710733115

[B6] Bandura A. (1997). Self-Efficacy: The Exercise of Control. New York, NY: Freeman

[B7] Barnett R. C. (1998). Toward a review and reconceptualization of the work/family literature. Gene. Soc. Gene. Psychol. Monographs 124, 125–184.

[B8] Biddle B. J. (1986). Recent developments in role theory. Annu. Rev. Sociol. 12, 67–92. 10.1146/annurev.so.12.080186.000435

[B9] Bogren M. Von Friedrichs Y. Rennemo Ø. Widding Ø. (2013). Networking women entrepreneurs: Fruitful for business growth? Int. J. Gender Entrepreneurship 5, 60–77. 10.1108/17566261311305210

[B10] Bruni A. Gherardi S. Poggio B. (2004), Doing gender, doing entrepreneurship: an eth-nographic account of intertwined practices. Gender Work Organization 11, 406–29. 10.1111/j.1468-0432.2004.00240.x

[B11] Brush C. Carter N. Gatewood E. Greene P. Hart M. (2005). The Diana International Report: Research on Growth Oriented Women Entrepreneurs and Their Businesses. Stockholm: ESBRI. 10.4337/9781845429942

[B12] Carlson D. S. Kacmar K. M. Wayne J. H. Grzywacz J. G. (2006). Measuring the positive side of the work-family interface: Development and validation of a work-family enrichment scale. J. Voc. Behav. 68, 131–164. 10.1016/j.jvb.2005.02.002

[B13] Confindustria (2020). Seconda Edizione dell'indagine Sugli Effetti Della Pandemia da Covid-19 per le Imprese Italiane. Available online at: https://www.confindustria.it/wcm/connect/12cc3799-1107-4a66-95f4-923cf34c2aab/Seconda+Edizione+Indagine+Covid_15-04-2020.pdf?MOD=AJPERES&CACHEID=ROOTWORKSPACE-12cc3799-1107-4a66-95f4-923cf34c2aab-n6IFscK (accessed June 2, 2020).

[B14] Cooper A. C. Gimeno-Gascon F. J. Woo C. Y. (1994). Initial human and financial capital as predictors of new venture performance. J. Business Ventur. 9, 371–395. 10.1016/0883-9026(94)90013-2

[B15] De Simone S. Agus M. Lasio D. Serri F. (2018). Development and validation of a measure of work-family interface. J. Work Org. Psychol. 34, 169–179. 10.5093/jwop2018a19

[B16] De Simone S. Priola C. (2015). “What's women's work? Work-family interface among women entrepreneurs in Italy,” in Handbook of Gendered Careers in Management: Getting in, Getting On, Getting Out, eds A. Broadbridge, and S. Fielden (Cheltenham: Edward Elgar), 390–408.

[B17] De Simone S. Priola V. (in press). “Who's that Girl?” The entrepreneur as a super(wo)man. Canad. J. Administ. Sci.

[B18] Del Boca D. Oggero N. Profeta P. Rossi M. (2020). Women's and men's work, housework and childcare, before and during COVID-19. Rev. Econ. Household 18, 1001–1017. 10.1007/s11150-020-09502-1 32922242 PMC7474798

[B19] DeMartino R. Barbato R. Jacques P. H. (2006). Exploring the career/achievement and personal life orientation differences between entrepreneurs and nonentrepreneurs: the impact of sex and dependents. J. Small Business Manage. 44, 350–368. 10.1111/j.1540-627X.2006.00176.x

[B20] Dijkhuizen J. Gorgievski M. van Veldhoven M. Schalk R. (2016). Feeling successful as an entrepreneur: a job demands—resources approach. Int. Entrepreneurship Manage. J. 12, 555–573. 10.1007/s11365-014-0354-z

[B21] Dijkhuizen J. Van Veldhoven M. Schalk R. (2014). Development and validation of the entrepreneurial job demands scale. Int. J. Knowledge Innovat. Entrepreneurship 2, 70–88.

[B22] Fahlén S. (2014). Does gender matter? Policies, norms and the gender gap in work-to-home and home-to-work conflict across Europe. Commun. Work Family 17, 371–391. 10.1080/13668803.2014.899486

[B23] Frone M. R. (2003). “Work-family balance,” in Handbook of Occupational Health Psychology, eds J. C. Quick and L. E. Tetrick (Washington, DC: American Psychological Association), 143–162.

[B24] GDA (2020). Global Data Analysis. Coronavirus (COVID-19) Executive Briefing. London: Global Data.

[B25] Greenhaus J. Powell G. (2003). When work and family collide: Deciding between competing role demands. Organ. Behav. Hum. Decis. Process. 90, 291–303. 10.1016/S0749-5978(02)00519-8

[B26] Grzywacz J. G. Butler A. B. (2005). The impact of job characteristics on work-to-family facilitation: testing a theory and distinguishing a construct. J. Occup. Health Psychol. 10, 97–109. 10.1037/1076-8998.10.2.97 15826221

[B27] Grzywacz J. G. Marks N. F. (2000). Reconceptualizing the work–family interface: an ecological perspective on the correlates of positive and negative spillover between work and family. J. Occup. Health Psychol. 5, 111–126. 10.1037/1076-8998.5.1.111 10658890

[B28] Guirdham M. (ed.). (1990). “Interaction planning and conclusion,” in Interpersonal Skills at Work (Wiltshire: Redwood Books), 399–412.

[B29] Hall D. T. (1990). Promoting work/family balance: an organization-change approach. Organ. Dyn. 18, 5–18. 10.1016/0090-2616(90)90060-3

[B30] Hill E. J. (2005). Work-family facilitation and conflict, working fathers and mothers, work-family stressors and support. J. Fam. Issues 26, 793–819. 10.1177/0192513X05277542

[B31] Hobfoll S. E. Johnson R. J. Ennis N. Jackson A. P. (2003). Resource loss, resource gain, and emotional outcomes among inner city women. J. Pers. Soc. Psychol. 84, 632–643. 10.1037/0022-3514.84.3.632 12635922

[B32] Hsu D. K. Wiklund J. Anderson S. E. Coffey B. S. (2016). Entrepreneurial exit intentions and the business-family interface. J. Business Ventur. 31, 613–627. 10.1016/j.jbusvent.2016.08.001

[B33] Istat (2019). I Tempi Della Vita Quotidiana - Lavoro, Conciliazione, Parità di Genere e Benessere Soggettivo, Rome: Istat.

[B34] Jennings J. E. McDougald M. S. (2007). Work-family interface experiences and coping strategies: implications for entrepreneurship research and practice. Acad. Manage. Rev. 32, 747–760. 10.5465/amr.2007.25275510

[B35] Kim J. L. S. Ling C. S. (2001). Work-family conflict of women entrepreneurs in Singapore. Women Manage. Rev. 16, 204–221. 10.1108/09649420110395692

[B36] Kinnunen U. Feldt T. Geurts S. Pulkkinen I. (2006). Types of work-family interface: well-being correlates of negative and positive spillover between work and family. Scand. J. Psychol. 47, 149–162. 10.1111/j.1467-9450.2006.00502.x 16542357

[B37] Koltai L. Geambasu R. Bakacsi-Saffer Z. Barna-Petróczi A. Zsár V. (2020). COVID-19 and Female Entrepreneurs Throughout Europe. Budapest: Hetfa Research Institute Ltd.

[B38] Kuratko D. F. Hornsby J. S. Naffziger D. W. (1997). An examination of owner's goals in sustaining entrepreneurship. J. Small Business Manage. 35, 24–33.

[B39] Lee N. Zvonkovic A. M. Crawford D. W. (2014). The impact of work-family conflict and facilitation on women's perceptions of role balance. J. Fam. Issues 35, 1252–1274. 10.1177/0192513X13481332

[B40] Loscocco K. Bird S. (2012). Gendered paths: Why women lag men in small business success. Work Occup. 39, 183–219. 10.1177/0730888412444282

[B41] Loscocco K. A. Robinson J. Hall R. H. Allen J. K. (1991). Gender and small business success: an inquiry into women's relative disadvantage. Soc. Forces 70, 65–85. 10.2307/2580062

[B42] McGee J. E. Peterson M. Mueller S. L. Sequeira J. M. (2009). Entrepreneurial self-efficacy: refining the measure, *Entrepreneurship Theory and Practice*, 33. 10.1037/t49726-000

[B43] Netemeyer R. G. Boles J. S. McMurrian R. (1996). Development and validation of work–family conflict and family–work conflict scales. J. Appl. Psychol. 81, 400–410. 10.1037/0021-9010.81.4.400

[B44] Noor N. (2004). Work-family conflict, work- and family-role salience, and women's well-being. J. Soc. Psychol. 144, 389–405. 10.3200/SOCP.144.4.389-406 15279329

[B45] Nurmi J.-E. Toivonen S. Salmela-aro K. Eronen S. (1997). Social strategies and loneliness. J. Soc. Psychol. 137, 764–777. 10.1080/00224549709595497 9414626

[B46] Osservatorio sull'imprenditoria femminile (2020). Welfare e Istruzione: 1 Impresa su 3 è guidata da Donne. Available online at: www.unioncamere.gov.it/download/10045.html (accessed March 22, 2020).

[B47] Peris-Ortiz M. Rueda-Armengot C. Osorio D. (2012). Women in business: entrepreneurship, ethics and efficiency. Int. Entrepreneurship Manage. J. 8, 343–354. 10.1007/s11365-011-0177-0

[B48] Poggesi S. Mari M. De Vita L. (2019). Women entrepreneurs and work-family conflict: an analysis of the antecedents. Int. Entrepreneurship Manage. J. 15, 431–454. 10.1007/s11365-017-0484-1

[B49] Powell G. N. Eddleston K. (2011). Work-family enrichment and entrepreneurial success: do female entrepreneurs benefit most? Acad. Manag. Proc. 2011, 1–6. 10.5465/ambpp.2011.65869189

[B50] Remuzzi A. Remuzzi G. (2020). COVID-19 and Italy: what next? Lancet 395, 1225–1228. 10.1016/S0140-6736(20)30627-9 32178769 PMC7102589

[B51] Rothbard N. P. (2001). Enriching or depleting? The dynamics of engagement in work and family roles. Administ. Sci. Q. 46:655. 10.2307/3094827

[B52] Ruderman M. N. Ohlott P. J. Panzer K. King S. N. (2002). Benefits of multiple roles for managerial women. Acad. Manage. J. 45, 369–386. 10.2307/3069352

[B53] Schaufeli W. B. Bakker A. B. (2004). Job demands, job resources, and their relationship with burnout and engagement: a multi-sample study. J. Organizational Behav. 25, 293–315. 10.1002/job.248

[B54] Shane S. Locke E. A. Collins C. J. (2003). Entrepreneurial motivation. Human Resource Manage. Rev. 13, 257–279. 10.1016/S1053-4822(03)00017-2

[B55] Shelton L. M. (2006), Female entrepreneurs, work–family conflict, and venture per- formance: new insights into the work–family interface. J. Small Business Manage. 44, 285–97. 10.1111/j.1540-627X.2006.00168.x.

[B56] Shelton L. M. Danes S. M. Eisenman M. (2008). Role demands, difficulty in managing work-family conflict, and minority entrepreneurs. J. Dev. Entrepreneurship 13, 315–342. 10.1142/S1084946708000983

[B57] Simpson M. Tuck N. Bellamy S. (2004). Small business success factors: the role of education and training. Educ. Train. 46, 481–491. 10.1108/00400910410569605

[B58] Unioncamere (2021). Le donne pagano il conto più salato della pandemia: il Covid azzera la crescita delle imprese femminili. Available online at: https://www.unioncamere.gov.it/download/10948.html (accessed January 30, 2021).

[B59] Van Praag C. M. Versloot P. H. (2007). What is the value of entrepreneurship? A review of recent research. Small Business Econ. 29, 351–382. 10.1007/s11187-007-9074-x

[B60] Von Gelderen M. Frese M. Thurik R. (2000). Strategies, uncertainty and performance of small business start-ups. Small Business Econ. 15, 165–181. 10.1023/A:1008113613597

[B61] Wach D. Stephan U. Gorgievski M. (2016). More than money: developing an integrative multi-factorial measure of entrepreneurial success. Int. Small Business J. Research. Entrepreneurship 34, 1098–1121. 10.1177/0266242615608469

[B62] Walker E. Brown A. (2004). What success factors are important to small business owners? Int. Small Business J. Research. Entrepreneurship 22, 577–594. 10.1177/0266242604047411

[B63] Welter F. (2004). The environment for female entrepreneurship in Germany. J. Small Business Enterprise Dev. 11, 212–221. 10.1108/14626000410537155

[B64] Xanthopoulou D. Bakker A. B. Demerouti E. Schaufeli W. B. (2007). The role of personal resources in the job demands-resources model. Int. J. Stress Manage. 14, 121–141. 10.1037/1072-5245.14.2.12133451042

[B65] Zhao X. Frese M. Giardini A. (2010). Business owners' network size and business growth in China: the role of comprehensive social competency. Entrepreneurship Region. Dev. 22, 675–705. 10.1080/08985620903171376

[B66] Zhu N. Zhang D. Wang W. Li X. Yang B. Song J.. (2020). A Novel coronavirus from patients with pneumonia in China, 2019. N. Engl. J. Med. 382, 727–733. 10.1056/NEJMoa2001017 31978945 PMC7092803

